# Editorial: Exploring neurotological health concerns post-COVID-19 infection

**DOI:** 10.3389/fneur.2025.1731001

**Published:** 2025-11-18

**Authors:** Alexandra E. Quimby

**Affiliations:** Department of Surgery, Division of Otolaryngology-Head & Neck Surgery, Dalhousie University, Halifax, NS, Canada

**Keywords:** COVID-19, long COVID, SARS-CoV-2, neurotology, sudden sensorial hearing loss, vestibular dysfunction

In the years since the onset of the COVID-19 pandemic, a multitude of downstream health effects of SARS-CoV-2 infection have come to be recognized. These long-term health effects are wide-ranging, involving anywhere from a single to multiple organ systems, and can have profound effects on the health and quality of life of previously infected individuals in the long-term post-viral recovery. “Long COVID” can affect global functioning, neurocognition, cardiovascular, respiratory, gastrointestinal, and metabolic health. Even years after the expiration of the COVID-19 Public Health Emergency, previously unknown effects of the virus are being increasingly recognized. Auditory and vestibular dysfunction following COVID-19 infection, though speculative in the early days of the pandemic, have come to greater light as evidence accumulates pertaining to these and a variety of other neurotologic sequelae. Reported neurotologic presentations in the context of SARS-CoV-2 infection include sensorineural hearing loss—particularly the acute form, sudden sensorineural hearing loss (SSNHL)—tinnitus, and vestibular disorders such as vestibular loss and benign paroxysmal positional vertigo (BPPV). For example, a large population-based cohort study in South Korea found an adjusted hazard ratio of 3.44 for any new hearing loss diagnosis among SARS-CoV-2 infected individuals compared to uninfected controls (1). Though this represented an otherwise high-quality study, the use of International Classification of Diseases (ICD) codes to define new hearing loss diagnoses can be biased in several ways, limiting the internal validity of the study findings. Many other studies of the effects of SARS-CoV-2 are similarly fraught with methodologic short fallings inherent to the study of emerging infectious disease agents. For example, studies showing elevated risk of BPPV during COVID-19 raise the question of whether this relationship is actually secondary to prolonged bedrest and immobility rather than viral infection itself. A 2021 systematic review by Almufarrij and Munro found at that time 56 published articles on patients developing audiovestibular symptoms following SARS-CoV-2 infection, the majority of which were of evaluated to be “fair” quality (2). Only five studies in this review were deemed “good” quality. Despite the accumulation of additional research in the years since, there remains a need for high-quality studies and rigorous assessment of the nature and nuances of these diseases in the context of SARS-CoV-2 infection.

Determining causality as to the effects of SARS-CoV-2 infection is a challenge met in the study of any infectious disease agent. In the face of an inability to gather evidence from well-controlled interventional studies, multiple complimentary lines of evidence are necessary to attribute health effects directly to microbial infection. Causal inference frameworks are drawn upon; mechanistic evidence that harkens back to Koch's postulates is necessary; and large, well-controlled observational study designs provide a primacy of evidence. Each of these modes of evidence has contributed to our understanding of COVID-19′s neurotologic manifestations to date. Even in a post-pandemic reality, achieving a comprehensive understanding of the virus' effects and the mechanisms by which these are exerted is crucial from a public health standpoint. Our population has unprecedented vulnerability to global pandemics.

A cornerstone of understanding the effects of SARS-CoV-2 infection on human health is precise characterization of disease presentation. A wide body of research ranging across medical specialty disciplines endeavors to do just this. Researchers studying the effects of the virus in the short- and long-term post-infection have elucidated nuances of its presentation and provided insight into how these can be measured and followed. Concerning the virus' neurotologic sequelae, Peron et al. provide important insights into the audiometric profile of SSNHL related to SARS-CoV-2 infection. Their prospective cohort study assessed patients presenting with SSNHL for COVID infection via PCR testing and symptomatology and found a higher incidence of bilateral simultaneous SSNHL among patients with COVID infection and severe symptoms. These findings have important implications for the work-up of patients presenting with bilateral SSNHL, given that a proportion of these may represent COVID-infected individuals. From a vestibular standpoint, Corrêa et al. demonstrate the feasibility of assessing long-term balance impairment following viral infection using smartphone-based apps. They used smartphone inertial sensors to evaluate balance in patients with long COVID, finding greater postural instability on static balance tasks in these patients compared to controls. Though this approach is not specific to the assessment of patients with a history of SARS-CoV-2 infection, the study provides insights into its applicability in this population. These types of studies play a pivotal role in achieving a comprehensive understanding of the virus' effects. With large segments of the global population suffering long-term effects of SARS-CoV-2 infection, this knowledge is ever important.

Complimentary and equally necessary to clinical observational research are mechanistic studies. SARS-CoV-2 has been shown to exert its multi-system effects through a combination of mechanisms. These include but are not limited to direct viral entry in cells of target tissues, vascular endothelial injury driving inflammation, thrombosis (which may drive multi-organ ischemic injury), and immune dysregulation. The latter two effects have been long-theorized as accounting for certain presentations of non-infectious related audiovestibular dysfunction (for example, autoimmune or steroid-responsive inner ear disease and possibly some cases of idiopathic sudden sensorineural hearing loss). Recent research studying inner ear cells collected from the vestibular end organs at the time of surgical labyrinthectomy have demonstrated that these cells express the membrane receptors necessary for SARS-CoV-2 infection (3). Additional work has demonstrated other possible mechanistic pathways for the neurotologic effects of the virus; for example, SARS-CoV-2 particles have been demonstrated in middle ear effusions following viral infection. Li et al. explore this phenomenon in their study included as part of this Research Topic.

Another class of negative health outcomes that emerged during the pandemic are those with possible links to COVID-19 vaccination rather than to infection itself. The most consistently recognized serious adverse effects of the vaccine are thrombosis and thrombocytopenia, both of which are rare events. Though many practicing otolaryngologists have anecdotal reports of patients experiencing sudden sensorineural hearing loss or vertigo following COVID vaccination, these of course are unsubstantiated and represent the lowest quality of evidence. The study “Comparison of the rates of emergent otologic adverse events following mRNA COVID-19 vs. influenza vaccination: a matched cohort analysis” by Munjal et al. rigorously addresses this potential association, with surprising results. This matched cohort study included a total of 42,859 individuals receiving the COVID-19 vaccine or any pre-pandemic influenza vaccine and found no difference between the two groups in the odds of any hearing loss, sudden hearing loss, tinnitus, or otalgia. While the odds of aural fullness were significantly higher among the COVID vaccinated cohort compared to the influenza vaccinated cohort, the odds of vertigo were significantly lower among COVID-vaccinated individuals.

To summarize, the neurotologic manifestations of SARS-CoV-2 infection and vaccination represent a still-rapidly evolving field at the intersection of clinical otology/neurotology and infectious diseases. Lessons from this and previous pandemics make it increasingly clear that both observational and mechanistic research are essential to delineate the pathways by which COVID-19 and its prevention strategies may affect human health, including the auditory and vestibular systems. Rigorous clinical and laboratory-based studies are key to distinguishing viral mechanisms of injury from coincidental associations. The contributions assembled in this Research Topic collectively advance our understanding of these complex relationships—ranging from clinical characterizations and diagnostic insights to explorations of underlying mechanisms—and highlight the importance of continued systematic inquiry to inform patient care and future public health preparedness.

## References

[1] Kim HJ Jeong S Kim K Lee JD Oh YH Suh MJ. Incidence of hearing loss following COVID-19 among young adults in South Korea: a nationwide cohort study. EClinicalMedicine. (2024) 75:102759. doi: 10.1016/j.eclinm.2024.102759 39175987 PMC11339059

[2] Almufarrij I Munro KJ. One year on: an updated systematic review of SARS-CoV-2, COVID-19 and audio-vestibular symptoms. Int J Audiol. (2021) 60:935–45. doi: 10.1080/14992027.2021.1896793 33750252

[3] Jeong M Ocwieja KE Han D Wackym PA Zhang Y Brown A . Direct SARS-CoV-2 infection of the human inner ear may underlie COVID-19-associated audiovestibular dysfunction. Commun Med. (2021) 1:44. doi: 10.1038/s43856-021-00044-w 34870285 PMC8633908

